# A case of adenocarcinoma of the rete testis accompanied by focal adenomatous hyperplasia

**DOI:** 10.1186/1746-1596-8-105

**Published:** 2013-06-24

**Authors:** Xu-Yong Lin, Juan-Han Yu, Hong-Tao Xu, Liang Wang, Chui-Feng Fan, Yang Liu, En-Hua Wang

**Affiliations:** 1 Department of Pathology, the First Affiliated Hospital and College of Basic Medical Sciences, China Medical University, Shenyang, 110001, China; 2 Institute of pathology and pathophysiology, China Medical University, Shenyang, 110001, China

**Keywords:** Adenocarcinoma, Rete testis, Adenomatous hyperplasia

## Abstract

**Abstract:**

Adenocarcinoma of the rete testis is very rare. There is still little knowledge about its etiology and pathogenesis. Herein, we present a case of rete testis adenocarcinoma in a 36-year-old Chinese male. The tumor was predominantly composed of irregular small tubules and papillary structures with cuboidal or polygonal cells. In peripheral area of the tumor, the remaining normal rete testis and adenomatous hyperplasia of the rete testis could also be seen, indicating the possible relationship between adenomatous hyperplasia and adenocarcinoma. In addition, the patient underwent a left hydrocelectomy because of the existence of hydrocele 3 years ago. But, it is unclear whether hydrocele and hydrocelectomy is its cause or just the early clinical presentation of the adenocarcinoma.

**Virtual slides:**

The virtual slide(s) for this article can be found here: http://www.diagnosticpathology.diagnomx.eu/vs/6757609119625499

## Background

Adenocarcinoma of the rete testis is a very uncommon malignancy with approximately 60 cases reported in the literatures [1]. Because of the rarity, its etiology and histogenesis is still unclear. It usually occurs in men older than 60 years, although the age can range from 17 to 91 years [2]. The clinical manifestation is not specific. The most common manifestation is painless scrotal swelling; the other uncommon signs include hydrocele, epididymitis and inguinal hernia [2]. The histologic diagnosis of this tumor is usually difficult. To date, the generally accepted histologic criteria proposed by Nochomovitz and Orenstein include the location of the tumor in the mediastinum of the testis rather than intraparenchymal, transition from normal epithelial structures to neoplastic structures in the rete testis, no evidence of teratoma, exclusion of any primary tumor of a distant site, lack of direct extension through the tunica and a predominantly solid gross appearance [3]. However, it is difficult for many tumors to meet all of the above criteria. Especially, it is often hard to see the transition from normal epithelial structures to neoplastic structures in the rete testis, as the tumor used to destroy the normal rete testis tissue. It is speculative that adenomatous hyperplasia of the rete testis may be the precursor lesion of adenocarcinoma [4,5]. Herein, we present a case of adenocarcinoma of the rete testis in a 36-year-old Chinese male. Histologically, tumor demonstrates the apparent transition from normal rete testis to adenomatous hyperplasia, at last to adenocarcinoma, suggesting the close relationship between the adenomatous hyperplasia and adenocarcinoma.

## Case presentation

### Clinical history

A 36-year-old male referred to our hospital for complaining of a painful swelling in the left testis 1 year ago. Physical examination demonstrated that the left testis apparently enlarged, and felt firm. Laboratory examination revealed values of serum alpha-fetoprotein (AFP), alkaline phosphatase (AP), CA19-9, CA125 and prostate specific antigen (PSA) were in normal level. Scrotal ultrasound revealed that there was an irregular, solitary mass about 7.5 × 4.3 × 4.0 cm in the lower region of the left testis. No lesions in other organs including lung, prostate and rectum were detected. The patient reported had undergone a hydrocelectomy for hydrocele and slight enlargement of the testis 3 years ago. However, after the first surgery, the testis still gradually enlarged, and increased in size rapidly for the past six months. A second surgery was then performed in our hospital. At surgery, there was a gray-yellow mass in the testis, and the testis with the mass was removed, and underwent diagnostic examination. According to the morphological and immunohistochemical findings, the tumor was diagnosed as an adenocarcinoma of the rete testis. Then the patient underwent BEP (bleomycin, etoposide and cisplatinum) chemical therapy two times. He was alive with no tumor recurrence or metastasis within 15 months of follow-up.

## Materials and methods

The resected specimens were fixed with 10% neutral-buffered formalin and embedded in paraffin blocks. Tissue blocks were cut into 4-μm slides, deparaffinized in xylene, rehydrated with graded alcohols, and immunostained with the following antibodies: cytokeratin (CK,AE1/AE3, 1:50, DAKO), cytokeratin 5/6 (CK 5/6, 1:200, DAKO), cytokeratin7 (CK7, 1:200, DAKO), Vimentin (1:200, DAKO), CD30 (1:100, DAKO), carcino embryonic antigen (CEA, 1:100, DAKO), α-Fetoprofein (AFP, 1:200, DAKO), human chorionic gonadotropin beta (HCG-β, 1:100, DAKO), thyroid transcription factor 1 (TTF-1, 1:100, DAKO), epithelial membrane antigen (EMA, 1:200, DAKO), Prostate Specific Antigen (PSA,1:100, Santa cruz), CA19-9 (1:100, Santa cruz), CA125 (1:100, Santa cruz), Calretinin (1:100, DAKO),α-inhibin (1:100, DAKO), PLAP (1:100, DAKO), CD117 (1:100, DAKO) and Ki67 (1:200, DAKO). Sections were stained with a streptavidin-peroxidase system (KIT-9720, Ultrasensitive TM S-P, MaiXin, China). The chromogen used was diaminobenzidine tetrahydrochloride substrate (DAB kit, MaiXin, China), slightly counterstained with hematoxylin, dehydrated and mounted. For the negative controls, the primary antibody was replaced with PBS. This study was prospectively performed and approved by the institutional Ethics Committees of China Medical University and conducted in accordance with the ethical guidelines of the Declaration of Helsinki.

## Results

### Gross features

Grossly, the testis was approximately 8.3 × 5.1 × 4.9 cm, was involved by a firm, irregular 7.1 × 4.2 × 4.1 cm tumor. The tumor was relatively well circumscribed, mainly located in the region of testicular hilum. The cut face of the tumor was grey-yellow or grey-white in color. The tunica of the testis was lost.

### Microscopic features

**Figure 1:**
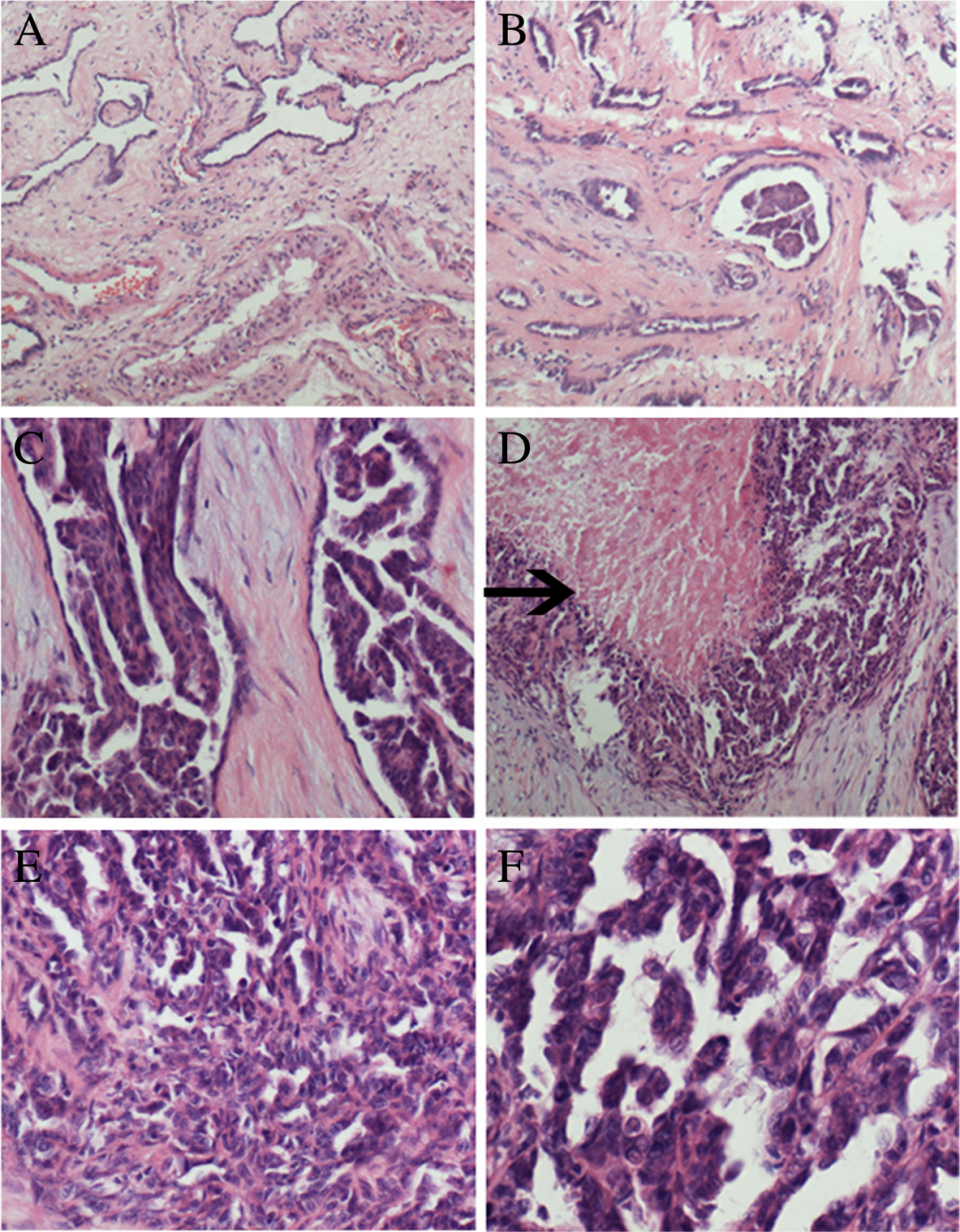
**Morphological change of the tumor. A**, The remaining normal rete testis tissue was present in the peripheral area of the tumor. **B**, The area comprising irregular tubules with highly collagenized stroma represented adenomatous hyperplasia of the rete testis. **C**, In the distended tubules, the cells formed apparent papillary patterns. **D**, The apparent necrosis (black arrow) was present in the papillary structure. **E**, The area was composed of irregular small tubules and complicated papillary structures with little collagenized stroma. **F**, The cells had marked cellular atypia with dark staining chromatin and conspicuous nucleoli.

Histologically, the tumor was mainly confined to testicular hilum. The tumor was predominantly composed of irregular small tubules and complicated papillary structures with cuboidal or polygonal cells. Focally, the cells were arranged into solid sheets or masses with apparent necrosis. Amidst the tumor cells, little fibrovascular stroma could be seen. The cells had marked cellular atypia, with dark staining chromatin and conspicuous nucleoli. The mitosis was very common. In the peripheral area of the tumor, the remaining normal rete testis could be seen. In addition, an area of adenomatous hyperplasia which consisted of irregular tubule also presented, the adenomatous hyperplasia was located within the normal rete testis. In several distended tubules, the papillary hyperplasia of epithelial cells could be seen (Figure 1).

### Immunohistochemistry

**Figure 2:**
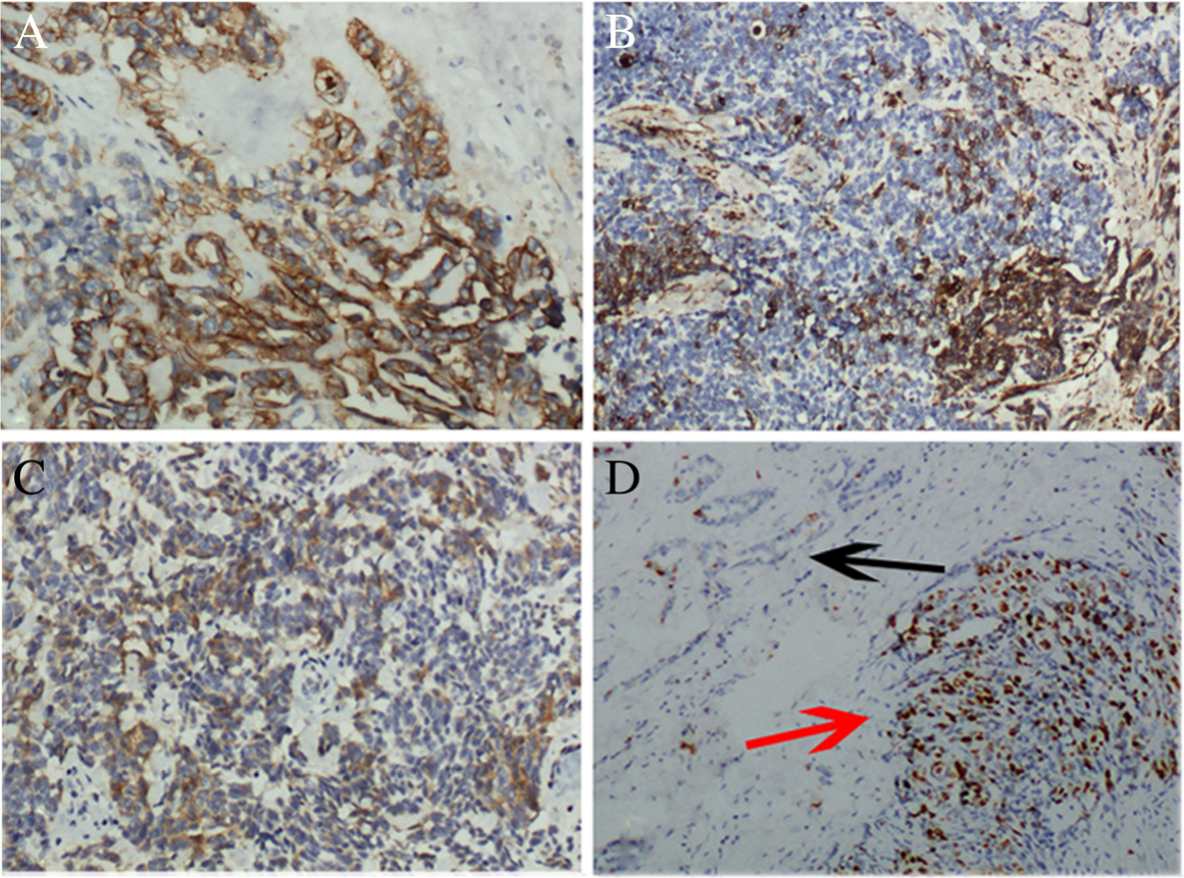
**Immunohistochemical staining of the tumor. A**, The cells were entirely positive for CK. **B**, The tumor cells were focally positive for Vimentin. **C**, Focal positive expression of CEA was present in the tumor cells. **D**, The area showed the transition from adenomatous hyperplasia (black arrow) to adenocarcinoma (red arrow). Ki67 index was less than 5% in adenomatous hyperplasia; in contrast, Ki67 index was approximately 40% in adenocarcinoma.

Immunohistochemical staining showed that the tumor cells including area of adenomatous hyperplasia were diffusely positive for EMA, CK, and CK7, focally positive for CEA and Vimentin, negative for CK 5/6, CD30 , AFP, HCG-β, TTF-1, PSA, CA19-9, CA125, Calretinin, PLAP, CD117 and α-inhibin. Ki67 index was approximately 40%, In contrast, in the area of adenomatous hyperplasia, the Ki67 proliferative index was less than 5% (Figure 2). According to the morphological and immunohistochemical findings, the tumor was diagnosed as an adenocarcinoma of the rete testis.

## Discussion

The rete testis is a relatively inconspicuous structure. Histologically, it is an anastomosing network consisted of delicate tubules. Adenocarcinoma of the rete testis is very rare, thus its etiology and histogenesis is still unclear [1]. It was reported that exposure to diethylstilbestrol could cause various degrees of papillary proliferation, hyperplasia of the epithelium and adenocarcinoma of the rete testis in the male mice [6]; while in humans no reports of hyperplasia and adenocarcinoma were attributed to exposure to diethylstilbestrol. Gruber H et al. reported a case of adenocarcinoma of the rete testis originated from a fireman with the history long-term exposure to chemical agents such as carbon dioxide, lead and fire extinguishing agent [5]. However, in our reported case, the patient works as a farmer, and he has not the history of long–term exposure to chemical drugs including diethylstilbestrol. The clinical manifestation of this tumor is variable. In addition to the painless scrotal swelling [7], the incidental findings include hydrocele, epididymitis and inguinal hernia [2]. In some cases, the history of hydrocele or chronic epididymitis exited several years before the presence of swelling [8,9]. In our case, 2 years before finding the swelling, the main sign was hydrocele. However, it is still unclear whether hydrocele or chronic epididymitis is the predisposing factor or the initial manisfection of this tumor. In addition, in several reported cases, the history of trauma also exited before the tumor diagnosis [10], indicating the prior trauma may be related to this tumor. In our case, the patient did not report the history of trauma, so the relationship between the trauma and the adenocarcinoma of the rete testis should be further testified.

The first case of adenomatous adenomatous hyperplasia of the rete testis was reported by Nistal and Paniagua in 1988 [11]. In the case reported by Gruber H et al., the preexisting adenomatous hyperplasia of the rete testis was found 10 years before the presence of the scrotal mass [5]. In diethylstilbestrol exposure mouse model, the lesions resembling adenocarcinoma as well as hyperplasia of the rete testis were found [6]. So, It is speculative that adenomatous hyperplasia of the rete testis may be the precursor lesion of adenocarcinoma. But, the direct transition from the hyperplasia of the rete testis to the adenocarcinoma is usually hard to see. To date, the generally accepted histologic criteria of the adenocarcinoma of the rete testis include the location of the tumor in the mediastinum of the testis, transition from normal epithelial structures to neoplastic structures in the rete testis, no evidence of teratoma, exclusion of any primary tumor of a distant site, lack of direct extension through the tunica and finally a predominantly solid gross appearance . In our case, some area main consists of the irregular tubules with abundant collagenized stroma. We think this area simply represents adenomatous hyperplasia of the rete testis (Figure 1B, C). In contrast, in the other area the tumor was predominantly composed of irregular small tubules and complicated papillary structures with little stroma. Focally, the cells were arranged into solid sheets or masses with apparent necrosis. This area represents the adenocarcinoma of the rete testis(Figure 1D-F). The apparent transition from normal rete testis to adenomatous hyperplasia and adenocarcinoma suggests the close relationship between them.

The reported immunohistochemical staining findings are not completely consistent [12-14]. In general, it is always positive for CK, and negative for AFP, CD30, HCG-β, PSA and α-inhibin, which is helpful to exclude the origin of prostate or germ cell. However, the expression of CEA, Vimentin, EMA and Calretinin does not appear uniform [5,15-17].

Although the diagnostic criteria seem to be well established, the correct diagnosis of this rare tumor is still a hard work. The differential diagnosis includes metastatic adenocarcinoma [18], malignant mesothelioma [19] and other uncommon tumors [20,21]. Metastatic adenocarcinoma can be ruled out based on the clinical history. Mesothelioma is the most challenging differential diagnosis. Mesothelioma arises in the tunica vaginalis, while the adenocarcinoma mainly involved the mediastinum of the testis. Immunohistochemically, mesothelioma usually positive for CK, Vimentin, Calretinin and CK5/6, and negative for CEA, while adenocarcinoma of the rete testis is usually positive for CK, and negative for CK5/6 [19]. Because of the variable immunostaining pattern for CEA, Vimentin and Calretinin in the latter, the diagnosis may be exceptionally difficult. In our case, the patient had undergone a hydrocelectomy before the presence of the swelling, so we can exclude the possibility of malignant mesothelioma. Furthermore, our case was immunopositive for EMA, CK, and CK7 focally positive for CEA and Vimentin, and immunonegative for CK 5/6 and Calretinin. We can also rule out malignant mesothelioma on the basis of the positive expression of CEA and negative expression of CK 5/6 and Calretinin.

Carcinoma of the rete testis is a highly aggressive tumor with a reported survival of 13% at 5 years [2]. Our case underwent chemical BEP therapy two times after the surgery. The patient was alive with no tumor recurrence or metastasis within 15 months of follow-up.

## Conclusion

Because of the rarity, etiology and histogenesis of adenocarcinoma of the rete testis is still not completely clear. Our reported case demonstrates the apparent transition from normal rete testis to adenomatous hyperplasia and adenocarcinoma. It suggests that the adenomatous hyperplasia may be the precancerous lesion of adenocarcinoma of the rete testis.

## Consent

Written informed consent was obtained from the patient for publication of this case report and accompanying images. A copy of the written consent is available for review by the Editor-in Chief of this Journal.

## Competing interests

The authors declare that they have no competing interests.

## Authors’ contributions

LXY participated in the histopathological evaluation, performed the literature review, acquired photomicrographs and drafted the manuscript. YJH and XHT carried out the immunohistochemical stains evaluation. WL FCH and LY conceived and designed the study. WEH gave the final histopathological diagnosis and revised the manuscript. All the authors read and approved the final manuscript.
